# Association between Work and Chronic Obstructive Pulmonary Disease (COPD)

**DOI:** 10.3390/jcm7100335

**Published:** 2018-10-09

**Authors:** H. F. van der Molen, G. J. de Groene, C. T. J. Hulshof, M. H. W. Frings-Dresen

**Affiliations:** Amsterdam UMC, Coronel Institute of Occupational Health, Netherlands Center for Occupational Diseases, Amsterdam Public Health Research Institute, P.O. Box 22660, 1100 DD Amsterdam, The Netherlands; g.j.degroene@amc.uva.nl (G.J.d.G.); c.t.hulshof@amc.uva.nl (C.T.J.H.); m.frings@amc.nl (M.H.W.F.-D.)

**Keywords:** chronic obstructive pulmonary disease (COPD), vapors, dusts, gases and fumes (VDGF), occupational disease, etiology

## Abstract

To support occupational physicians in their assessment and notification of occupational diseases, diagnostic registration guidelines are developed with information about associations between work-related risk factors and diseases. The objective of this review of systematic reviews is to examine whether work-related risk factors are associated with chronic pulmonary obstructive disease (COPD). We searched the electronic database of Medline for systematic reviews published between 1 January 2009 and 20 June 2017. Reviews were included when COPD was assessed by data on lung function and when work-related exposures to vapors, dusts, gases, or fumes (VDGF) were described. One author selected studies and extracted data; two authors assessed study quality using A MeaSurement Tool to Assess systematic Reviews (AMSTAR). In all eight systematic reviews included, various exposures to vapors, dusts, gases, and fumes (VGDF) at work are associated with COPD. Two-thirds of the included studies are cross-sectional and show a high heterogeneity in population, setting, and mostly self-reported-exposures. Two high-quality reviews (AMSTAR score ≥ 9) including meta-analyses show associations and excess risk of COPD for work-related general exposure to VDGF with a summary odds ratio of 1.4 (95% confidence interval (CI) 1.19–1.73) and to inorganic dust with a mean difference in predicted forced expiratory volume in one second (FEV_1_) of −5.7% (95% CI: −8.62% to −2.71%). Exposure to VGDF at work is associated with a small but increased risk of COPD. More detailed workplace measurements of specific VGDF are warranted to gain an insight into dose–response relationships.

## 1. Introduction 

Worldwide, chronic obstructive pulmonary disease (COPD) is a common condition with reported prevalences of up to 12% [1,2,3]. Smoking is the main factor in causing COPD [1,4,5], but evidence from systematic reviews indicates that occupational exposures may also contribute, e.g., [6,7,8]. Biological plausibility for reported associations between occupational exposure to respiratory irritants and COPD is supported by toxic inhalation studies [6,9].

The knowledge about the role of occupational exposures in the development of COPD has not been without controversy [10] and is under debate in various national occupational compensation schemes for occupational diseases [11]. An overview of systematic reviews, summarizing evidence of pooled estimates of associations between vapors, dusts, gases, and fumes (VDGF) and COPD is lacking for the clinical and occupational health care setting.

Diagnostic guidelines, based on systematic reviews, contain information about associations between work-related risk factors and diseases [12]. These guidelines are helpful in supporting occupational physicians in their assessment and notification of occupational diseases. Extending the search period of Omland et al. [6] and with the availability of multiple systematic reviews in Medline, we aimed to summarize reported associations between work and COPD. The objective of this review is to assess whether work-related risk factors are associated with COPD based on information from available systematic reviews.

## 2. Methods

Systematic reviews were included when outcome data were described in terms of assessed COPD with lung function and exposure to vapors, dusts, gases, or fumes (VDGF) among a working population. All types of exposure assessment were eligible for inclusion: self-reports (survey or interview), expert assessment, job exposure matrices (JEMs) and environmental measurements. No additional criteria were formulated regarding latency between exposure and COPD or adjustment for confounders. 

We searched the electronic databases of Medline for systematic reviews posted between 1 January 2009 and 20 June 2017 (see Table A1). Our PICO was: P = working population; I/C = exposed/less or none exposed to defined exposure categories; O = COPD. 

Titles and abstracts were screened by one review author (G.J.d.G.), and in case of doubt checked by another (H.F.v.d.M.), to identify potentially relevant systematic reviews. The full texts of potential systematic reviews were assessed for eligibility against the inclusion criteria. Data was extracted by one review author (G.J.d.G.) and checked by another (H.F.v.d.M.). Data variables extracted from each systematic review were: author; number and type of included study designs; number of participants; exposure measurements; case definition of COPD; effects.

The methodological quality of the included studies was assessed independently by two review authors (G.J.d.G. and H.F.v.d.M.), both using the AMSTAR (A MeaSurement Tool to Assess systematic Reviews) criteria [13]. In case of disagreement, a third review author was consulted. In total, 11 criteria for quality assessment were considered: (1) provision of an a priori design; (2) duplicate study selection and data extraction; (3) performance of a comprehensive literature search; (4) inclusion of grey literature; (5) provision of a list of included and excluded studies; (6) provision of characteristics of the included studies; (7) assessment of the quality of the included studies; (8) appropriate scientific quality in formulating conclusions; (9) appropriate use of methods to combine findings from studies; (10) assessment of publication bias; and (11) reporting of conflicts of interest. Each criterion was scored with ‘yes’, ‘no’, ‘cannot answer’, or ‘not applicable’. The number of ‘yes’ scores could thus range between 0 and 11. On this basis, the included studies were classified as high quality (‘yes’ score 9 to 11), moderate quality (5 to 8) and low quality (0 to 4).

A descriptive analysis of all studies was performed and summarized. All four review authors (G.J.d.G., H.F.v.d.M., C.T.J.H., M.H.W.F.-D.) discussed the qualitative synthesis.

## 3. Results 

### 3.1. Selected Studies

**Figure 1:**
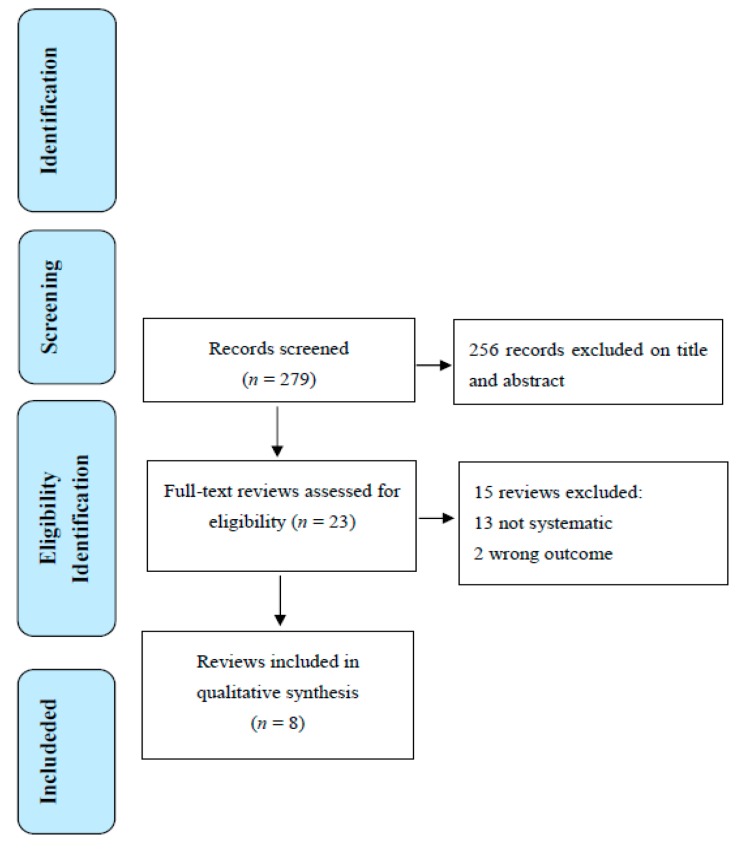
PRISMA (Preferred Reporting Items for Systematic Reviews) flow diagram of the selection of reviews.

In total, 279 references were retrieved from Medline (see Figure 1). The full texts of 23 potentially eligible reviews were then examined, with eight found to meet the inclusion criteria. Four reviews also provided a meta-analysis. Fifteen reviews were excluded because these were not systematic (13) or had the wrong outcome (2).

### 3.2. Methodological Quality

The methodological quality of the included systematic reviews ranges from meeting four of the eleven criteria to meeting nine of them (see Table 1). The criteria most frequently not met are provision of an a priori design, inclusion of grey literature, and provision of a list of included and excluded studies. Two-thirds of the included studies in the systematic reviews are cross-sectional and show a high heterogeneity in population, setting and mostly self-reported-exposures.

### 3.3. Summary of Included Reviews

In all the systematic reviews, various work exposures to VDGF are associated with COPD [6,7,8,14,15,16,17,18]. In particular, production industries, mining, farming and the construction industry and inorganic dust [6,7,8,15,16,17], organic dust or biological agents [6,18] are reported as having an association with COPD.

Two high-quality reviews with meta-analyses [14,15] show associations with COPD for work exposure to VDGF with a summary odds ratio adjusted for smoking of 1.4 (95% confidence interval (CI) 1.19–1.73) and to inorganic dust with a mean difference in predicted forced expiratory volume in one second (FEV_1_) of −5.7% (95% CI: −8.62% to −2.71%). In a summary estimate of the two longitudinal study in the review of Brüske et al. [15], adjusted for age and smoking, a mean annual decline of FEV_1_ of 6.3 mL higher was reported for biopersistent granular dust exposed participants compared to low/no exposed participants.

Two moderate-quality reviews with meta-analyses and adjusted or stratified for smoking [7,8] reported associations with COPD for biological dust, mineral dust, and gases/fumes [8] and respirable quartz dust among granite workers, concrete workers, automotive foundry, and tunneling workers [7].

The two other studies of moderate quality showed associations between construction workers and COPD, whereof among never-smokers in one study [16], and between organic and inorganic/mineral dust exposure with dose–response relationship [6].

Two low-quality reviews [17,18] reported associations with COPD for ammonia, cement (organic) dust, chlorine, cleaning agent, mustard gas, diesel exhaust, environmental (tobacco) smoke, isocyanate, sulphur dioxide, endotoxins, mites, ammonia, hydrogen sulfide, and work environments like construction, swine confinement, (livestock) farming, foundry, and metallurgical industry.

## 4. Discussion

All systematic reviews show associations between VDGF and COPD. Two high-quality reviews with meta-analyses show an excess risk of COPD for work exposure to general VDGF of 40% [14] and a 6% decrease in predicted FEV_1_ for inorganic dust [15].

As in our review, the majority of occupational COPD studies focus on dusty environments but no large differences in risk estimates were found for the most common occupational airborne pollutants [19]. Depending on the prevalence of exposure to VGDF, the reported occupational contribution to COPD burden varies between 3% for exposure to VGDF [20] and 20% or even higher estimates for specific work environments [10]. From a pathological point of view, smoking is part of VGDF and therefore a confounder in the association between work and COPD. Smoking and VGDF at work have a synergistic effect on COPD, however VGDF without smoking shows attributable risk estimates between 27–53% [11]. So, for individual smoking and non-smoking workers VGDF at work increase the likelihood of COPD [11].

In order to manage and prevent COPD, it is necessary to identify which specific type of VDGF exposure at work contributes most to the condition. Occupational exposures are thought to occur not only in the major basic industries but also in serviced industries with different processes [10]. Besides reducing work exposures, smoking cessation remains a key intervention strategy for chronic respiratory diseases [21].

Strengths of this review are its inclusion of systematic reviews, with four of them also incorporating meta-analyses and having the highest AMSTAR score [7,8,14,15]. All the moderate and high-quality reviews corrected for smoking or stratified their results into smokers and non-smokers and indicating an association between VGDF and COPD. Our review of systematic reviews did not examine the effect of smoking alone on COPD. Limitations are the fact that the majority of the included studies have a cross-sectional design and are often based on self-reported exposures. Higher risk estimates are reported for self-reported exposure to VDGF by comparison with JEM-based exposure to VGDF (pooled OR 1.9 versus 1.1) [19] and the number of cross-sectional study designs highlights the need for higher-quality studies. More detailed environmental measurements of specific VDGF by occupational health and safety professionals and greater awareness among employers and workers of the potential risks could stimulate the implementation of preventive measures to reduce work-related COPD.

In summary, exposure to VGDF at work is associated with a small but increased risk of COPD. More detailed workplace measurements of specific VGDF are warranted to gain an insight into dose–response relationships.

## Figures and Tables

**Table 1:** Summary of findings from systematic reviews for associations between work and chronic obstructive pulmonary disease (COPD)

Author	Number and Type Studies *	Number of Participants	Exposure and Measurements	COPD Case Definition **	Effects	Quality (AMSTAR)
Ryu 2015 [14]	*n* = 11 Meta-analyses CS: 7 CC: 4	26,959	**VGDF** JEM, Expert assessment, and Self-report	GOLD, FEV_1_/FVC < 70% FEV_1_/FVC < LLN	**VGDF** No exposure versus exposure Odds Ratio = 1.43 (95% CI 1.19–1.73)	High (9/11)
Brüske 2013 [15]	*n* = 27 Meta-analyses CS: 17	39 to 3336	**Inorganic exposure**	FEV_1_/FVC < various FEV_1_ reduction	**Inorganic dust** No/Low versus high exposure: Mean difference FEV_1_: 160 mL (95% CI: −270 mL to −40 mL) Mean difference of FEV_1_ in % predicted: −5.7% (−8.6% to −2.7%) FEV1/FVC < 70% increased by 7% per 1 mg·m^−3^ respirable dust	High (9/11)
Alif 2016 [8]	*n* = 5 Meta-analyses L:1; CS: 4	1017 to 4267	**Biological dust** **Mineral dust** **Gases/fumes** JEM and Self-report	FEV_1_/FVC < 70% FEV_1_/FVC < LLN	**Biological dust** No effect **Mineral dust** No exposure versus exposure FEV1/FVC < 70%: OR = 1.15, 95% CI 1.04–1.27 **Gases and fumes ** High vs low exposure OR = 1.21, 95% CI 1.02–1.44	Moderate (8/11)
Brüske 2014 [7]	*n* = 10 Meta-analyses L: 2; L&CS:1; CS: 3	90 to 417	**Respirable quartz dust** Environmental	FEV_1_ reduction	**Inorganic quartz dust** No/low exposed versus exposed: Mean difference FEV_1_: −4.6% (95% CI: −7.18% to −2.06%) Standardized mean difference FEV_1_: −0.27 (−0.40 to −0.14) Standardized mean difference FEV_1_/FVC: −0.41 (−0.54 to −0.28)	Moderate (7/11)
Omland 2014 [6]	*n* = 59 L:22, CS: 37	7332	**VGDF** JEM, Expert assessment, Self-report **(In)organic dust**: Environmental measurements and Self-report	Gold 2+ FEV1/FVC FEV1/FVC < various	**VGDF** 22 out of 25 population based studies **Inorganic, mineral dust** 12 out of 15 industrial or occupational studies in welding (2), coal (2), coke (1), asphalt (1), silica (2), tunnel work (1), cadmium (1), glass bangle (1), and bleach (1) **Organic dust, biological agents** 17 out of 19 industrial or occupational studies in cotton (4), flax (1), jute (1), farming (4), grain (2), wood (3), rubber (2); 3 out four 4 industrial or occupational studies in endotoxin (3)	Moderate (6/11)
Borup 2017 [16]	*n* = 12 L:7; CC: 2; CS:3;	114 to 228,614	**Construction** Job titles in construction	Fatal COPD GOLD II–IV FEV_1_/FVC < LLN	**Inorganic dust** 9 out of 12 studies in construction workers	Moderate (6/11)
Baur 2012 [17]	*n* = 20 Not specified	Not specified	**VGDF** Measurements not specified	Lung function testing Not specified	**VGDF** Agents and professional work-sites (number of studies): ammonia (1), cement dust (4), chlorine (1), cleaning agent (1), mustard gas (1), diesel exhaust (2), environmental tobacco smoke (1), isocyanate (1), smoke (1), sulphur dioxide (1), construction (3), swine confinement (1), farming (1), foundry (1), metallurgical industry (1)	Low (4/11)
Fontana 2017 [18]	*n* = 14 L:1; CS:13	52 to 5420	**Farming ** Environmental, JEM, Self-report	Gold 2+ FEV_1_/FVC < various	**Organic dust, biological agents** Organic dusts, endotoxins, mites, ammonia, hydrogen sulfide and livestock farmers	Low (4/11)

* Type of studies: L = longitudinal; CC = case control; CS = cross-sectional. ** Case definition COPD: LNN = lower limits of normal based on the fifth percentile of the distribution of expected values of lung function; FEV_1_ = forced expiratory volume in one second; FVC = forced vital capacity ratio; GOLD = Global Initiative for Chronic Obstructive Lung Disease definition; staging of the severity of COPD in GOLD is based on the reduction in FEV_1_ (as percent predicted); VGDF: vapors, dusts, gases, and fumes; JEM: job exposure matrices; CI: confidence interval; OR: odds ratio.

## Appendix A

**Table A1:** Search strategy.

1. exp pulmonary disease, chronic obstructive/
2. (COPD or chronic airflow limitation or AECOPD or COAD or Chronic Obstructive Pulmonary Disease or Chronic Obstructive Airway Disease or Chronic Obstructive Lung Disease or Chronic Airflow Obstruction * or chronic bronchitis or pulmonary emphysema or lung emphysema).ab,kf,ti.
3. or/1–2 [COPD]
4. Women, Working/or exp Occupations/or exp Work/or Workplace/or exp Occupational Diseases/or exp Rehabilitation, Vocational/or Occupational Health/or Sick Leave/or Absenteeism/or workers’ compensation/or exp Employment/or exp Occupational Exposure/
5. (worka* or worker? or workg* or working or workp* or work capacity or work disabilit* or work abilit* or “at work” or work exposure or work place or work productivity or work related or workers or job* or employee or staff or personnel or occupation or occupations or occupational or outdoor work* or day shift* or night shift* or shift work* or vocational rehabilitation or sick leave or absenteeism or presenteeism or "return to work" or vocational reintegration or employment or work status or industries).ab,kf,ti.
6. or/4–5 [work related]
7. 3 and 6
8. limit 7 to “reviews (best balance of sensitivity and specificity)”
9. 8
10. limit 9 to year = “2009–Current”

COPD: chronic obstructive pulmonary disease; AECOPD: acute exacerbations of chronic obstructive pulmonary disease; COAD: chronic obstructive airway disease; *: truncation; ab: abstract, kf: author keywords, ti: title.
